# Sirtuin1 in Spinal Cord Injury: Regulatory Mechanisms, Microenvironment Remodeling and Therapeutic Potential

**DOI:** 10.1111/cns.70244

**Published:** 2025-02-06

**Authors:** Jinze Li, Shengyu Cui, Yanqiu Li, Can Zhang, Chao Chang, Fengzeng Jian

**Affiliations:** ^1^ Department of Neurosurgery Xuanwu Hospital, Capital Medical University Beijing China; ^2^ Spine Center China International Neuroscience Institute (CHINA‐INI) Beijing China; ^3^ Lab of Spinal Cord Injury and Functional Reconstruction China International Neuroscience Institute (CHINA‐INI), Xuanwu Hospital, Capital Medical University Beijing China; ^4^ Center for Integrative Medicine, Beijing Ditan Hospital Capital Medical University Beijing China; ^5^ Department of Neurosurgery The First Hospital of Hebei Medical University Shijiazhuang China

**Keywords:** deacetylase, microenvironment, regulatory mechanism, Sirt1, spinal cord injury

## Abstract

**Background:**

Spinal cord injury (SCI) is a complex central nervous system disorder characterized by multifaceted pathological processes, including inflammation, oxidative stress, programmed cell death, autophagy, and mitochondrial dysfunction. Sirtuin 1 (Sirt1), a critical NAD^+^‐dependent deacetylase, has emerged as a promising therapeutic target for SCI repair due to its potential to protect neurons, regulate glial and vascular cells, and optimize the injury microenvironment. However, the regulatory roles of Sirt1 in SCI are complex and challenging, as its effects vary depending on activation timing, expression levels, and cell types.

**Methods:**

A systematic literature review was conducted using PubMed, Scopus, and Web of Science to identify studies investigating Sirt1 in SCI. Relevant publications were analyzed to synthesize current evidence on Sirt1's mechanisms, therapeutic effects, and challenges in SCI repair.

**Results:**

Sirt1 exerts broad regulatory effects across diverse pathological processes and cell types post‐SCI. It promotes neuronal survival and axonal regeneration, modulates astrocytes and microglia to resolve inflammation, supports oligodendrocyte‐mediated myelination, and enhances vascular endothelial function. Proper Sirt1 activation may mitigate secondary injury, whereas excessive or prolonged activation could impair inflammatory resolution or disrupt cellular homeostasis. This review highlights Sirt1 activation as potential therapies, but challenges include optimizing spatiotemporal activation and addressing dual roles in different cell types.

**Conclusion:**

Targeting Sirt1 represents a viable strategy for SCI repair, given its multifaceted regulation of neuroprotection, immunomodulation, and tissue remodeling. However, translating these findings into therapies requires resolving critical issues such as cell type‐specific delivery, precise activation timing, and dosage control. This review provides a theoretical foundation and practical insights for advancing Sirt1‐based treatments for SCI.

## Graphical abstract

Sirtuin 1 (Sirt1), a key NAD^+^‐dependent deacetylase, has become a leading therapeutic target for spinal cord injury (SCI) repair due to its regulation of multiple pathological processes, including inflammation, oxidative stress, and cell death. Sirt1 not only protects and regenerates neurons but also modulates astrocytes, microglia, oligodendrocytes, and vascular endothelial cells to optimize the SCI microenvironment. However, its regulatory mechanisms are intricate, varying based on activation timing, expression levels, and cell types.

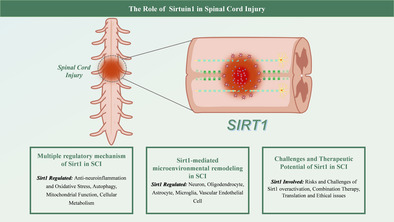

## 1 Introduction

Spinal cord injury (SCI) ranks as a leading cause of lifelong disability worldwide [1]. Characterized by high rates of disability and mortality, this disease presents significant public health challenges and places a substantial financial strain on both the societal and individual levels [2, 3]. The neuronal damage and disruption of neural circuits caused by SCI are often irreversible, resulting in severe motor, sensory, and autonomic dysfunctions in patients [4]. Typically, SCI encompasses a primary injury stage‐often due to direct physical impact proportional to the force applied and a secondary injury stage [5], which involves a cascade of events such as bleeding, inflammation, ion imbalance, and oxidative stress, leading to programmed cell death and demyelination [6, 7]. These processes not only aggravate the extent of the primary injury but also hinder neural repair and functional recovery [8]. Despite significant advances in scientific research, effective treatments for SCI remain a major challenge, underscoring the critical need for discovering new methods to repair SCI [9].

Currently, various therapeutic strategies for SCI are being actively investigated. Stem cell‐based approaches show promise in neural regeneration and tissue repair but face challenges in cell survival, differentiation control, and functional integration [10, 11]. Nanotechnology‐based drug approaches can achieve targeted delivery; however, their long‐term biosafety and delivery complexity, mainly due to the blood–brain barrier challenge and high cost and production difficulty, still require further study [12, 13]. CRISPR‐based gene editing offers precise genetic modification but raises concerns about off‐target effects and the efficiency of delivery systems [14, 15]. In this context, targeting endogenous molecular regulators that influence multiple pathological processes has emerged as an attractive alternative strategy.

Sirtuins, a class of endogenous histone deacetylases that rely on nicotinamide adenine dinucleotide (NAD^+^), have attracted particular attention as potential therapeutic targets [16, 17, 18]. Among them, Sirtuin1 stands out due to several unique characteristics: (1) as an evolutionarily conserved molecule, it is widely present across different species and has well‐established safety profiles, supporting its biological compatibility; (2) Sirt1 has unique advantages through their ability to simultaneously regulate multiple biological processes including metabolism, oxidative stress and inflammatory responses [19, 20, 21]. The versatility of Sirt1 also underscores its potential in SCI repair, where it regulates multiple mechanisms affecting neurons, astrocytes, oligodendrocytes, microglia, and vascular endothelial cells, thus facilitating tissue repair [22, 23, 24]. For instance, Sirt1 reduces oxidative stress and apoptosis, enhancing neuronal survival and axon regeneration by deacetylating specific transcription factors [25, 26]. In astrocytes and microglia, Sirt1 regulates their reactivity and changes their polarity, reducing the neuroinflammatory response and promoting neural repair [27, 28]. Furthermore, Sirt1 maintains the integrity of the blood–brain and blood‐spinal cord barriers (BSCB), shielding neural tissues from inflammatory damage [29]. (3) its activation can be easily achieved through both pharmacological agents (such as Sirt1 activators) and physiological approaches. Despite increasing evidence suggesting that modulating Sirt1 holds potential therapeutic benefits for SCI repair, The function of the Sirt1 signaling pathway in SCI and the mechanisms involved have not been thoroughly clarified in a systematic manner.

This review will systematically explore the mechanism of Sirt1 in SCI, focusing on its extensive regulatory mechanisms across different pathological processes and cell types under spinal cord microenvironmental imbalances. By summarizing the challenges and prospects of Sirt1 in the treatment of SCI, this review seeks to establish a theoretical foundation for future research on Sirt1 pathway modulation and its potential role in SCI repair.

## 2 The Multiple Cellular Regulatory Mechanism of Sirt1

Sirt1 is a NAD^+^‐dependent deacetylase known for its broad regulatory functions in cellular processes such as inflammation, oxidative stress, autophagy, metabolism, and mitochondrial function [19, 30, 31]. Its structure features a conserved catalytic core and multiple regulatory domains, allowing interactions with a broad array of substrates such as histones and non‐histone proteins [32]. This structural versatility underpins Sirt1's ability to modulate gene expression and cellular functions across different cell types.

Sirt1 plays a multifaceted role in the repair process following SCI. It offers neuroprotection by moderating inflammatory responses, thus limiting secondary injuries and enhancing cell survival [33]. Sirt1 also bolsters the cellular antioxidant defense, reducing oxidative stress—a significant factor in SCI‐induced cellular damage [34]. Moreover, it plays a key role in maintaining mitochondrial function, regulating autophagy, and preserving metabolic homeostasis, all vital for cellular recovery and tissue repair [35, 36].

**FIGURE 1:**
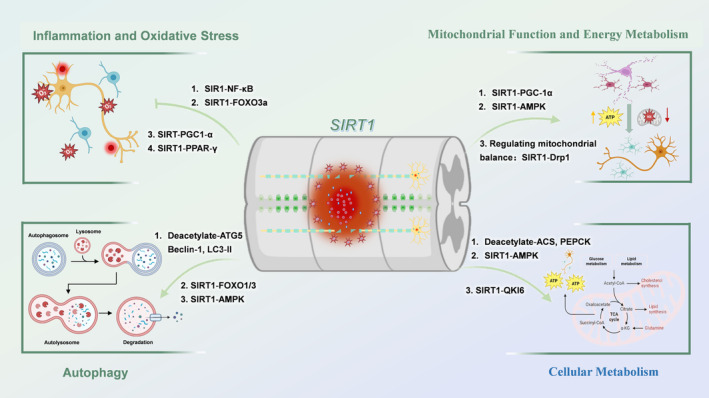
The multiple cellular regulatory mechanism of Sirt1. Sirt1 influences various cellular processes following SCI primarily through deacetylation, which prevents inflammatory responses and reduces oxidative stress. Additionally, Sirt1 activates autophagy pathways, enhancing SCI repair and providing neuroprotection. It plays a crucial role in regulating mitochondrial function and energy metabolism, maintaining energy balance. Moreover, Sirt1 significantly impacts cellular metabolism, including glucose and lipid metabolism, providing essential energy support for tissue repair and reconstruction post‐injury. ACS, Acyl‐CoA Synthetase; AMPK, AMP‐Activated Protein Kinase; ATG5, Autophagy‐Related 5; Drp1, Dynamin Related Protein 1; FOXO3a, Forkhead box O3; NF‐κB, nuclear factor‐kappa‐light‐chain‐enhancer of activated B‐cells; PEPCK, Phosphoenolpyruvate Carboxykinase; PGC1α, peroxisome Proliferator‐Activated Receptor Gamma Coactivator 1‐Alpha; PPAR‐γ, Peroxisome Proliferator‐Activated Receptor Gamma; QKI6, Quaking 6; SCI, spinal cord injury; Sirt1, sirtuin1.

The capacity of Sirt1 to regulate such varied mechanisms underscores its integral role in SCI repair (Figure 1). By coordinating multiple cellular processes, Sirt1 not only alleviates the pathological effects of spinal cord injury but also promotes nerve regeneration and functional recovery. This positions Sirt1 as a promising therapeutic target for promoting spinal cord repair outcomes.

### 2.1 Sirt1‐Regulated Anti‐Inflammatory and Oxidative Stress Pathways

Sirt1 plays a crucial role in modulating inflammatory responses and oxidative stress processes post‐SCI through its function as a NAD^+^‐dependent deacetylase. Specifically, studies have demonstrated that Sirt1 activation reduces pro‐inflammatory cytokine levels by 40%–50% and suppresses the expression of oxidative stress‐related proteins by 1–2‐fold within 24–48 h post‐injury [28, 34]. These processes are particularly critical in the acute and subacute phases of SCI, where uncontrolled inflammation and oxidative damage can lead to extensive secondary injury. The regulation of Sirt1 mitigates these reactions by targeting several critical signaling pathways [37].

One of the primary mechanisms through which Sirt1 regulates inflammation is by inhibiting the nuclear factor‐kappa B (NF‐κB) pathway, a central mediator of inflammatory responses. In the acute phase of SCI, the activation of NF‐κB triggers the release of pro‐inflammatory cytokines, leading to tissue damage and neuronal death. Sirt1 reduces the activity of NF‐κB by deacetylating its p65 subunit, leading to lower levels of pro‐inflammatory cytokines [38, 39]. This regulation is crucial for limiting the initial inflammatory cascade and preventing excessive tissue damage. Multiple studies have shown that various small molecule compounds, including Resveratrol, Tetramethylpyrazine, and Astragaloside IV can directly or indirectly activate Sirt1, leading to the downregulation of NF‐κB activity and the release of fewer inflammatory mediators [27, 33]. Furthermore, Sirt1 is believed to indirectly impact the formation and activation of the NLRP3 inflammasome, also via NF‐κB modulation, thus diminishing the generation of cytokines including IL‐1β and IL‐18 and exerting further anti‐inflammatory effects [40, 41]. The regulation of the NLRP3 inflammasome is particularly important during the subacute phase of SCI, as its activation can lead to pyroptosis and sustained inflammation. These results have positive implications for regulating the imbalance of the inflammatory microenvironment after SCI.

In addition to the NF‐κB pathway, Sirt1 also interacts with the Forkhead box O (FOXO) transcription factors, particularly FOXO3a, to foster the expression of genes involved in antioxidant stress processes [42]. During the acute and subacute phases of SCI, oxidative stress leads to mitochondrial dysfunction and cellular damage. Sirt1's deacetylation of FOXO3a enhances the expression of antioxidant enzymes like MnSOD and CAT, and anti‐inflammatory cytokines such as IL‐10, contributing to a more balanced inflammatory response [42, 43, 44]. This mechanism is crucial for regulating the intertwined dynamics of oxidative stress and inflammation in SCI pathology. This upregulation of antioxidant enzymes is crucial for protecting neurons and glial cells from oxidative damage, particularly in the early injury phase when oxidative stress peaks.

Moreover, Sirt1 enhances the activity of PPAR‐γ through deacetylation, boosting the expression of anti‐inflammatory genes. The PPAR‐γ pathway represents another critical mechanism through which Sirt1 modulates the post‐SCI inflammatory response. The activation of PPAR‐γ suppresses the production of pro‐inflammatory cytokines, reducing the inflammatory response [45]. Sirt1 also activates PGC‐1α, which works in tandem with PPAR‐γ to increase the expression of antioxidant enzymes and anti‐inflammatory pathways [46, 47]. Activation of the SIRT1/PGC‐1α pathway has been shown to promote and reduce neuroinflammation in animal models of nerve injury, alleviating mechanical abnormal pain [48]. Similarly, this synergistic action also contributes to the reduction of inflammation following SCI. The coordinated activation of PPAR‐γ and PGC‐1α by Sirt1 creates a powerful anti‐inflammatory and antioxidant effect, which is essential for limiting tissue damage and promoting repair after SCI.

Meanwhile, the anti‐inflammatory effects of Sirt1 exhibit distinct patterns across different SCI models and injury phases. In contusion models, the acute phase features rapid inflammatory cell infiltration and dramatic elevation of pro‐inflammatory cytokines (such as TNF‐α, IL‐1β, IL‐6), where Sirt1 activation primarily suppresses the early inflammatory cascade and reduces neutrophil infiltration [49]. During the subacute phase, as the inflammatory response evolves, Sirt1 plays crucial roles in modulating macrophage/microglial responses, reducing inflammatory mediator production, and initiating tissue repair processes [50]. In the chronic phase, Sirt1's effects gradually shift toward promoting long‐term inflammatory resolution and tissue remodeling, particularly through enhanced M2 microglial polarization and increased production of anti‐inflammatory factors such as IL‐10 and TGF‐β [51]. In comparison, complete transection SCI models exhibit distinctive inflammatory profiles characterized by complete disruption of neural circuits and more extensive tissue damage. Furthermore, the spatial distribution of inflammation shows a unique pattern, with inflammatory responses not only at the injury epicenter but also extending significantly to adjacent segments [52]. The activation of Sirt1 demonstrates distinct temporal effects across different phases following spinal cord transection. During the acute phase, its primary function is to contain and limit inflammatory spread to neighboring spinal segments. As the injury progresses to the subacute phase, Sirt1 takes on a crucial regulatory role in modifying the inflammatory microenvironment at the transection site, which is essential for potential tissue bridge formation. In the chronic phase, Sirt1 contributes to creating a more permissive environment that may support axonal regeneration attempts across the lesion gap [25, 53]. Understanding these model‐specific inflammatory patterns and Sirt1's differential regulatory roles is crucial for developing targeted therapeutic strategies for different types of SCI.

Overall, Sirt1 serves as a pivotal regulator in SCI, orchestrating a complex network of inflammatory and oxidative stress signaling events that help mitigate inflammation and facilitate spinal cord repair. Through its simultaneous regulation of multiple pathways including NF‐κB, FOXO3a, PPAR‐γ, and PGC‐1α, Sirt1 effectively coordinates the inflammatory and oxidative stress responses after SCI, creating an environment more conducive to tissue repair and functional recovery. Understanding these pathways not only elucidates the molecular underpinnings of SCI recovery but also highlights Sirt1 as a promising therapeutic target for modulating inflammation in SCI.

### 2.2 Sirt1‐Mediated Autophagy

Autophagy is a highly conserved intracellular degradation process that contributes to maintain cellular homeostasis, responding to stress, and promoting tissue repair [54, 55]. In the pathological process of SCI, autophagy serves as a crucial protective mechanism, helping to remove damaged organelles, protein aggregates, and cellular debris generated during both primary and secondary injury phases. The activation of Sirt1 can enhance autophagy flux by about twofold through increasing the LC3‐II/LC3‐I ratio and upregulating key autophagy proteins (ATG5, Beclin‐1) by 1.5‐fold [49, 56, 57].

First, Sirt1 can directly deacetylate key autophagy‐related proteins, enhancing their activity and promoting the formation of autophagosomes [58, 59, 60]. This regulation is particularly important in the early phase of SCI, where efficient clearance of damaged cellular components can significantly impact neuronal survival. Studies have demonstrated that hyperbaric oxygen therapy significantly upregulates Sirt1 post‐SCI, promoting an increase in pro‐autophagy formation proteins such as ATG5, Beclin‐1, and LC3‐II. However, this effect can be reversed by EX‐527, a Sirt1 inhibitor, illustrating the regulatory influence of Sirt1 on autophagy [49].

One of the primary mechanisms by which Sirt1 regulates autophagy is through the deacetylation of FOXO1 and FOXO3. This action leads to the increased expression of essential autophagy genes, such as Atg7 and LC3 [61]. Additionally, Sirt1 synergizes with AMPK to further regulate autophagy. On the contrary, the activation of AMPK leads to an elevation in NAD^+^ concentrations, further activating Sirt1 and upregulating autophagy‐related genes [62]. Furthermore, Sirt1 exerts an indirect activation Effect on AMPK through the deacetylation of Liver Kinase B1, which, once activated, promotes the expression of autophagy genes through phosphorylation pathways [35, 57]. This interplay between AMPK and Sirt1 establishes a positive feedback loop that significantly boosts autophagy efficiency following SCI.

In summary, Sirt1 is crucial in the pathophysiology and recovery of SCI by finely modulating the autophagy mechanism. In the acute phase, Sirt1‐mediated autophagy helps limit secondary damage by efficiently removing damaged cellular components. During the subacute and chronic phases, this regulated autophagy becomes essential for cellular remodeling and axonal regeneration, processes that are crucial for functional recovery. Moreover, Sirt1's regulation of autophagy in different cell types—including neurons, astrocytes, and oligodendrocytes—contributes to comprehensive tissue repair after SCI. Understanding the Sirt1‐autophagy axis in SCI deepens insight into the molecular pathology of SCI and offers a robust theoretical foundation for developing novel therapeutic strategies.

### 2.3 Sirt1 in Mitochondrial Function and Energy Metabolism

In the pathophysiological process of SCI, mitochondrial dysfunction and energy metabolism disorders reduce ATP production by 70% and increase ROS generation by threefold [63, 64]. Following the primary mechanical trauma of SCI, a series of secondary injury cascades are initiated, where mitochondrial dysfunction serves as a central pathological hub that connects multiple injury mechanisms including calcium overload, oxidative stress, and apoptotic signaling. Sirt1 is central to maintaining mitochondrial homeostasis and regulating energy metabolism, which is crucial for the repair process of SCI [65]. It restores mitochondrial membrane potential by 40% and upregulates mitochondrial transporter proteins by 55%, thereby promoting functional recovery after SCI [34, 36].

Sirt1 enhances mitochondrial biogenesis and function through the deacetylation and activation of PGC‐1α, a pivotal modulator of mitochondrial transcriptional activity. During the acute phase of SCI, mechanical injury directly disrupts mitochondrial membrane integrity and electron transport chain function, leading to rapid ATP depletion and increased ROS production. In this context, the regulation of Sirt1 affects both the quantity and quality of mitochondria, vital for sustaining cellular energy supply and combating oxidative stress post‐SCI [66]. Recently, a study showed that Caffeic acid phenethyl ester (CAPE) significantly improved mitochondrial membrane potential and reversed the decrease of mitochondrial protein TOM20 in mice with SCI by activating the Sirt1‐ PGC‐1α axis, which effectively preserved mitochondrial function by maintaining membrane potential and protein import machinery, thereby reducing neuronal death associated with energy failure [34].

The pathological progression of SCI involves complex alterations in mitochondrial dynamics. Sirt1 can also regulate mitochondrial dynamic balance, affecting the fusion and fission process of mitochondria, thereby maintaining the integrity and functional stability of the mitochondrial network [67, 68]. After SCI, mitochondrial fusion and fission need to be maintained in a certain state of balance to prevent further aggravation of cell damage. Excessive mitochondrial fission, triggered by elevated calcium levels and oxidative stress, results in mitochondrial fragmentation, cristae disruption, and eventual release of pro‐apoptotic factors. Mitochondrial dysfunction often leads to energy metabolism disorders and cell apoptosis [69, 70]. Research, including findings by Zhong et al. [36], shows that Sirt1, activated by melatonin post‐SCI, significantly inhibits Drp1‐mediated mitochondrial fission. The Sirt1‐Drp1 signaling axis can balance the fusion and fission processes of mitochondria, maintain mitochondrial dynamic balance, and avoid mitochondrial dysfunction and cell apoptosis caused by excessive mitochondrial fission.

Notably, Sirt1 and AMP‐activated protein kinase (AMPK) also synergistically regulate mitochondrial function and energy metabolism, forming a metabolic checkpoint that responds to energy stress conditions [71, 72]. After SCI, tissues are often in a state of inadequate energy supply and enhanced oxidative stress [70]. The disruption of blood supply and mitochondrial dysfunction creates a severe bioenergetic crisis, characterized by decreased ATP levels, impaired calcium homeostasis, and accumulated metabolic waste products. The synergistic effect of Sirt1‐AMPK regulates energy metabolism, helps damaged cells maintain energy balance, reduces oxidative stress damage to mitochondria, and prevents cell apoptosis, thereby exerting neuroprotection [57].

In summary, Sirt1's role in regulating mitochondrial function and energy metabolism is central to the repair and recovery processes in SCI. Through coordinated regulation of mitochondrial biogenesis, dynamics, and energy metabolism, Sirt1 addresses multiple aspects of mitochondrial dysfunction in SCI, from maintaining membrane potential and reducing ROS production to preserving energy homeostasis and preventing apoptotic cascade activation. By supporting mitochondrial health and energy balance, Sirt1 not only sustains the vitality of nerve cells but also promotes their repair and regeneration, presenting a promising therapeutic target for SCI treatment.

### 2.4 The Role of Sirt1 in Cellular Metabolism

Sirt1, highly conserved NAD^+^‐dependent deacetylase, plays a vital role in regulating cell metabolism. Following SCI, the initial mechanical trauma triggers an immediate metabolic crisis, characterized by ATP depletion, ion gradient disruption, and metabolic enzyme dysfunction. This acute metabolic disturbance initiates a cascade of secondary injury mechanisms that further compromise cellular function and survival. This molecule is involved in regulating multiple metabolic processes such as glucose metabolism, lipid metabolism, and energy metabolism [73, 74, 75].

In glucose metabolism, the primary mechanical injury disrupts spinal cord blood flow, leading to immediate glucose and oxygen deprivation in the injury epicenter. This initiates a pathological cascade where Sirt1 deacetylates and activates key enzymes in glucose metabolism, such as acetyl‐CoA synthetase in the glycolysis pathway and phosphoenolpyruvate carboxykinase involved in the gluconeogenesis pathway, serving as a crucial adaptive response to meet cellular energy demands under stress conditions [76, 77]. This mechanism maintains the energy supply of neurons and glial cells in the context of SCI. Moreover, Sirt1 activates the AMPK signaling pathway, enhancing glucose uptake and utilization [78]. The Sirt1‐AMPK axis functions as a metabolic sensor, responding to decreased ATP levels by upregulating glucose transporters and glycolytic enzymes. Studies have demonstrated that Rhodiola crenulata can significantly exert neuroprotective effects through the activation of the tricarboxylic acid (TCA) cycle via the Sirt1‐AMPK axis, thereby favorably meeting the energy demands of damaged neural tissue [79].

The pathophysiology of SCI involves extensive membrane damage and myelin disruption, placing significant demands on lipid metabolism for repair processes. The function of Sirt1 encompasses the regulation of lipid metabolism, where it regulates various transcription factors and enzymes involved in lipid processing [80, 81]. Related studies have shown that Sirt1 can deacetylate Quaking 6 (QKI 6), enhancing the triglyceride synthesis, which not only provides essential building blocks for membrane repair but also serves as an alternative energy substrate when glucose metabolism is impaired [47]. Interestingly, changes in lipid metabolism influence the expression of Sirt1. Cui et al. [26] discovered that inhibiting the synthesis of 20‐HETE offers a promising approach to alleviate mitochondrial dysfunction and neuronal apoptosis, primarily through the upregulation of the SIRT1/PGC‐1α signaling pathway. Furthermore, fatty acid supplementation, including Omega‐3 polyunsaturated fatty acids, has been observed to upregulate Sirt1, inhibit neuronal apoptosis, and confer neuroprotective effects [82].

In the context of SCI, Sirt1 is essential for reducing apoptosis, oxidative damage, and maintaining cellular energy balance by regulating multiple metabolic pathways, thereby enhancing the regeneration and repair capacity of tissues. The ability of Sirt1 to simultaneously regulate glucose utilization, lipid metabolism, and mitochondrial function positions it as a central metabolic regulator in the injury response. This metabolic regulation not only contributes to protect neurons and other glial cells, but also provides the necessary energy support for the remodeling of the damaged area.

## 3 Sirt1‐Mediated Microenvironmental Remodeling in Spinal Cord Injury

Sirt1 serves a crucial neuroprotective function in spinal cord injury (SCI), impacting a wide array of neural cell types. It modulates the functions of neurons [36, 83], astrocytes [27, 84], oligodendrocytes [85, 86], microglia [34, 51, 87], and vascular endothelial cells [29, 88, 89] through its deacetylase activity, promoting repair mechanisms following injury [90, 91].

**FIGURE 2:**
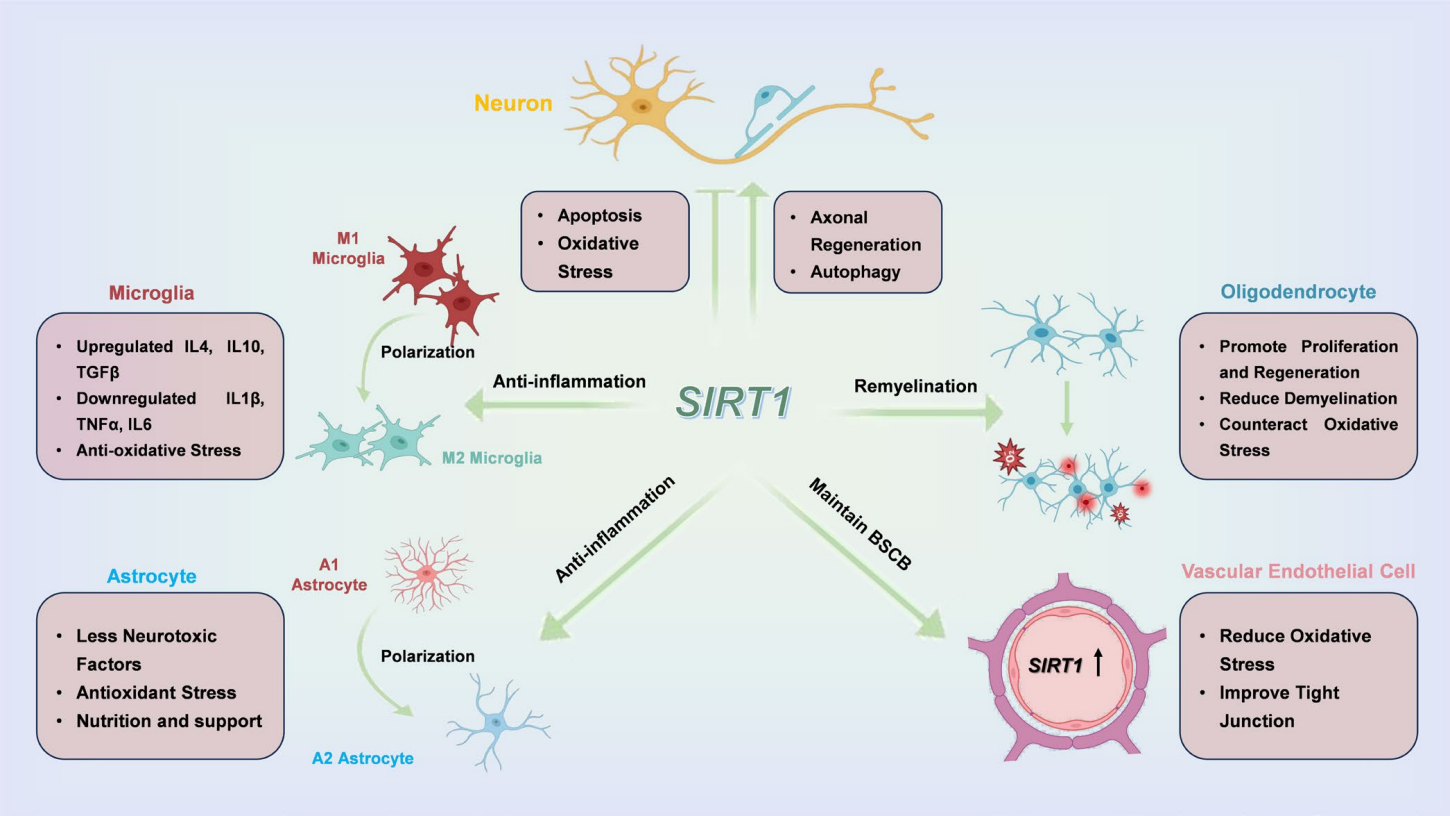
Regulatory effects of Sirt1 on multiple neural cells after SCI. After SCI, the regulation of Sirt1 can protect neurons from inflammation and oxidative stress in multiple pathways to promote neuronal survival, while facilitating axonal regeneration to achieve functional restoration. In addition, Sirt1 plays a vital role in promoting oligodendrocyte regeneration, along with reducing demyelination. It also shows a positive role in regulating the transition of microglia and astrocytes from a pro‐inflammatory to an anti‐inflammatory phenotype, which is important for alleviating the inflammatory microenvironment after injury. Sirt1 is crucial for maintaining endothelial cell function and alleviating BSCB disruption after SCI. BSCB, Blood‐Spinal Cord Barrier; IL10, Interleukin 10; IL1β, Interleukin 1 Beta; IL4, Interleukin 4; IL6, Interleukin 6; SCI, spinal cord injury; Sirt1, Sirtuin 1; TGFβ, Transforming Growth Factor Beta; TNFα, Tumor Necrosis Factor Alpha.

In terms of regulating neurons, Sirt1 promotes axonal regeneration and mitigates apoptosis, enhancing neuronal survival and repair capabilities [25, 36]. In oligodendrocytes, it stimulates proliferation and aids in the repair of myelin, essential for effective nerve signal transmission [85]. Sirt1 also reduces inflammatory damage by regulating the polarization and inflammatory response of microglia and astrocyte [27, 51]. Moreover, in vascular endothelial cells, Sirt1 contributes to the maintenance of BSCB integrity by modulating vascular endothelial cell function and facilitates the recovery process following SCI [29]. Sirt1's comprehensive regulation of these neural cell types underscores its pivotal role in SCI repair. The extensive influence of Sirt1 on these cell types highlights its critical role in orchestrating key processes such as neuroprotection, axon regeneration, glial function, inflammation management, and vascular remodeling. This coordination is vital for fostering functional recovery after SCI (Figure 2).

The multiple regulatory effects of Sirt1 not only contribute to restore neurological function, but also enhance the overall repair potential of SCI [92]. A deeper understanding of the effects of Sirt1 on various neural cell types will not only help to elucidate the complex pathological mechanisms of SCI, but also provide an important basis for the development of comprehensive therapeutic strategies targeting Sirt1.

### 3.1 Sirt1‐Mediated Neuroprotection and Axonal Regeneration

Sirt1 plays a critical role in neuroprotection and axon regeneration after SCI. Specifically, Sirt1 activation enhances neuronal survival rate by twofold through inhibiting the expression of apoptosis‐related proteins (such as Caspas3 and Bax, etc.) by 50% and promotes axon regeneration, which improves functional recovery scores by 40%–50% in standardized behavioral tests [22, 45, 93].

After SCI, neurons are often faced with multiple threats such as oxidative stress, inflammation, and disrupted energy metabolism [54]. Sirt1 regulates multiple pathways through its deacetylase activity, thereby inhibiting apoptosis. Research has indicated that melatonin inhibits neuronal apoptosis and activates autophagy after SCI by upregulating the Sirt1‐mediated AMPK pathway, thereby providing neuroprotection [35, 93]. Additionally, Sirt1 enhances the activity of PGC‐1α, boosting antioxidant gene expression, improving neuronal resistance to oxidative stress, reducing neuroinflammation, and preserving neuronal integrity [34]. In the acute phase of SCI, these mechanisms are critical for reducing secondary damage and protecting surviving neurons.

Secondly, the role of Sirt1 in axonal regeneration is equally striking. The molecular mechanisms underlying Sirt1's regulation of axonal regeneration are complex and multifaceted. Recent epigenetic research using a zebrafish model of SCI reveals Sirt1's pivotal role in axonal regrowth through modulating the HIPPO pathway. By deacetylating Dnmt1, Sirt1 leads to the hypomethylation of the Yap1 promoter, enhancing axonal regeneration capabilities [25]. This epigenetic modulation establishes a permissive molecular environment that facilitates axon regrowth. Additionally, a study discovered that overexpression of Sirt1 in spinal motoneurons induces mTOR‐independent autophagy, fostering a growth‐permissive state that enhances motor axon regeneration following nerve injury. The Sirt1/HIF1α‐autophagy axis was identified as a crucial pathway supporting axonal regeneration. Through this pathway, Sirt1 mediates the clearance of inhibitory debris and ensures adequate energy supply for axon extension, thereby creating favorable conditions for regeneration [53].

The molecular mechanisms underlying Sirt1's regulation of axonal regeneration also involve multiple interconnected signaling pathways. A crucial mechanism through which Sirt1 promotes axonal regeneration involves its regulation of the PI3K/Akt signaling pathway. Studies have demonstrated that Sirt1 activation leads to enhanced phosphorylation of Akt, which subsequently activates multiple downstream effectors critical for axonal growth [94]. Specifically, Sirt1 activates PI3K, leading to increased phosphorylation of Akt. The activated Akt then phosphorylates downstream targets including mTOR and GSK‐3β. The phosphorylation of mTOR enhances local protein synthesis in growth cones and regulates the assembly of microtubules and actin filaments, which are essential for axonal elongation [95]. Meanwhile, Akt‐mediated inhibitory phosphorylation of GSK‐3β reduces its negative regulation on several microtubule‐associated proteins, thereby promoting microtubule stability and axon assembly in the regenerating neurons [96]. Another potential mechanism promoting axonal regeneration is the Sirt1‐mediated regulation of p38 MAPK signaling. Specifically, Sirt1 directly deacetylates p38 MAPK, which induces conformational changes in p38 MAPK and reduces the binding affinity of upstream kinases (such as MKK3/6) to its phosphorylation sites (Thr180/Tyr182), thereby attenuating p38 MAPK phosphorylation and its kinase activity [97]. In the pathological context of SCI, hyperactivated p38 MAPK acts as a crucial mediator of secondary injury by amplifying inflammatory responses through increased pro‐inflammatory cytokine production, triggering oxidative stress via enhanced NADPH oxidase activation, and promoting neuronal apoptosis through upregulation of pro‐apoptotic proteins [98, 99]. These pathological processes create a hostile microenvironment that severely impairs axonal regeneration. Furthermore, activated p38 MAPK induces the expression of axon growth inhibitors, particularly chondroitin sulfate proteoglycans (CSPGs) [100, 101]. The Sirt1‐mediated deacetylation of p38 MAPK effectively interrupts these detrimental signaling cascades, thereby attenuating neuroinflammation, oxidative stress, and neuronal death while reducing the expression of axon growth inhibitors. This comprehensive modulation of the injury microenvironment is beneficial to axonal extension and functional recovery after SCI. Moreover, emerging evidence suggests that Sirt1 promotes axonal regeneration by negatively regulating the Notch signaling pathway, which is a critical inhibitor of axonal growth after SCI [102]. Mechanistically, Sirt1 deacetylates and inactivates the Notch intracellular domain (NICD), thereby suppressing the transcription of Notch target genes such as Hes1 and Hey1 that normally restrict axonal regeneration. The inhibition of Notch signaling by Sirt1 activation has been shown to enhance growth cone formation and axonal extension after injury [103]. Additionally, Sirt1‐mediated suppression of Notch signaling reduces astrogliosis and glial scar formation, creating a more permissive environment for axonal regeneration [104, 105]. This regulation appears to be particularly important during the subacute and chronic phases of SCI when excessive glial scarring can impede axonal regrowth.

Sirt1's influence extends beyond direct axonal growth to encompass synaptic plasticity and circuit reorganization after SCI. Current research indicates that Sirt1 plays a vital role in modulating synaptic proteins and influencing dendritic spine morphology, which are essential elements for establishing new neural circuits after injury [106, 107]. These synaptic modifications are particularly crucial during the chronic phase of SCI when circuit reorganization determines functional recovery outcomes [108, 109]. The coordinated regulation of axonal regeneration by Sirt1 is further supported by its effects on myelination. Through its regulation of oligodendrocyte differentiation and survival, Sirt1 facilitates proper myelination of regenerating axons. Studies have demonstrated that Sirt1 activation increases the expression of myelin‐associated proteins and enhances oligodendrocyte survival [85, 86]. This synchronized regulation of axonal growth and myelination by Sirt1 creates an optimal microenvironment for successful axonal regeneration and functional recovery.

Overall, Sirt1 plays a comprehensive regulatory role in neuronal protection and axonal regeneration through multiple signaling pathways, which not only enhances neuronal survival, but also promotes nerve regeneration after injury, providing a new perspective and potential therapeutic strategy for repair after SCI.

### 3.2 Sirt1 in Oligodendrocyte Regeneration and Myelin Repair

Oligodendrocytes (OL) are crucial for forming myelin sheaths around axons, which ensures rapid nerve conduction, thereby maintaining the normal function of the central nervous system [110]. After SCI, oligodendrocyte precursor cells differentiate into mature myelin‐forming cells, actively participate in myelin regeneration and repair at the site of injury, and balance the demyelination and remyelination processes [111]. These cells not only support the survival and functional recovery of axons, but also support the nutrient supply of neurons through the renewal of myelin sheaths, thereby promoting functional repair and regeneration after SCI [112]. Sirt1, as a key epigenetic regulator, is crucial for oligodendrocyte regeneration and the repair of myelin [17]. This molecule regulates the function of oligodendrocytes through multiple mechanisms and is of great significance for myelin reconstruction.

Research indicates that Sirt1 facilitates the deacetylation of retinoblastoma in the Rb/E2F1 complex in mice with neonatal brain injury, which significantly enhances OL proliferation and OL regeneration [113]. Furthermore, there is evidence that mice overexpressing Sirt1 exhibit significantly less demyelination and axonal damage in immune‐mediated spinal cord injury compared to control mice. The underlying mechanism may be Sirt1‐mediated increases in brain‐derived neurotrophic factor (BDNF) levels [85]. In models of white matter injury, Astragaloside IV can promote the survival of oligodendrocytes and increase the number of mature oligodendrocytes by upregulating Sirt1/Nrf2 signaling to counteract oxidative stress [86]. Moreover, in immune‐mediated spinal demyelinating disease, elevated Sirt1 levels corresponded with increased oligodendrocyte and reduced cell death, suggesting a critical regulatory role for this molecular in this nerve cell [114].

Taken together, Sirt1 plays a potential role in regulating oligodendrocyte function and myelin repair to promote neural repair after injury.

### 3.3 Sirt1 in Astrocyte Reactivity

Astrocytes are the main supporting cells in the central nervous system, constituting approximately 40%–50% of all neural cells [115]. They respond quickly after SCI and participate in the repair process by secreting inflammatory factors and forming glial scars [116]. However, excessive reactive astrocyte activation often leads to extensive scar formation, hindering nerve regeneration. Sirt1, as an NAD^+^‐dependent deacetylase, decreases GFAP expression and lowers pro‐inflammatory cytokine production by 50% through deacetylation of multiple transcription factors and histones. In addition, Sirt1 activation also reduced the expression of astrocyte A1 marker by nearly 50% and upregulated the expression of A2 marker by approximately twofold [27, 84].

First, Sirt1 is involved in regulating the phenotypic transition of reactive astrocytes. Toxic A1 astrocytes, as a hallmark of SCI, play a destructive role by secreting neurotoxic factors, inducing apoptosis of neurons and oligodendrocytes, and promoting inflammatory responses, thereby aggravating the injury and hindering the recovery of SCI [117]. Recent research has shown that through Sirt1 activation, the combination of tetramethylpyrazine and astragaloside IV reduces the proportion of detrimental A1‐type astrocytes and increases the beneficial A2‐type astrocytes, significantly ameliorating the disordered astrocyte polarization after SCI [27]. Another study showed that resveratrol, a Sirt1 agonist, markedly mitigated the activation of astrocytes and the secretion of pro‐inflammatory mediators in the spinal cord by inhibiting the FoxO1‐AQP5 axis, significantly improving chronic constriction injury‐induced neuropathic pain [118]. Besides, Sirt1 enhances the antioxidant capacity of astrocytes. By activating the Nrf2 signaling pathway, Sirt1 enhances the expression of antioxidant enzymes in astrocytes, such as superoxide dismutase (SOD). In vitro studies have indicated that the overexpression of the nuclear factor of activated T cells (NFAT), which leads to Sirt1‐Nrf2 upregulation, enhances astrocytes' resistance to oxidative stress and protects against damage from oxygen–glucose‐serum deprivation/restoration [119]. Similarly, related in vivo studies have also demonstrated that combination therapy with Botulinum Toxin and minocycline, through activating Sirt1, can attenuate SCI‐induced neuropathic pain by suppressing neuroinflammation and oxidative stress triggered by activated astrocytes in the spinal cord [84].

These investigations have revealed a significant association between Sirt1 and astrocytes, highlighting the role of Sirt1 in modulating astrocytic functions. It provides important support for the repair of SCI by regulating astrocyte polarity and inhibiting post‐injury oxidative stress and neuroinflammation.

### 3.4 Sirt1‐Regulated Microglial Polarization and Inflammation

Sirt1, as an important epigenetic regulatory factor, plays a crucial role in the functional regulation and polarization of microglia [120]. It affects the activation state, polarization direction, and inflammatory response of microglia through multiple mechanisms, thus having a profound impact on the pathological and repair process of SCI. Current studies demonstrate that it modulates microglial function and polarization with specific effects: reducing M1 markers by 50%, increasing M2 marker expression by 1.5‐fold, and decreasing pro‐inflammatory cytokine production by 45% [34, 51]. These changes correlate with a reduction in local inflammation and improved tissue repair.

In contrast, Sirt1 is involved in microglia‐mediated neuroinflammatory responses. Asiatic acid can reduce the production of pro‐inflammatory factors (such as TNF‐α and IL‐1β) derived from BV2 microglia by upregulating Sirt1, while promoting the release of anti‐inflammatory mediators, such as IL‐10 and TGF‐β [121]. Additionally, Chen et al. [50] indicated that inhibition of histone deacetylase 3 promoted Sirt1‐mediated Nrf2 deacetylation and its nuclear translocation, which significantly reduced SCI‐induced microglial activation and alleviated neuroinflammation. Sirt1's influence extends to the Wnt/β‐catenin signaling pathway in microglia. After treatment of LPS‐treated microglia with Sirt1 agonists, β‐catenin transcriptional activity decreased in a dose‐dependent manner. In vivo experimental results also suggest that Sirt1 may inhibit microglial activation by downregulating Wnt/β‐catenin signaling after SCI, thereby exerting a neuroprotective effect [122].

On the contrary, Sirt1 plays a critical role in regulating the polarization of microglia. Related studies have discovered that the administration of Sirt1 agonists into wild‐type SCI mice promoted the polarization of microglia to M2 type (anti‐inflammatory type) while inhibiting the polarization of M1 type (pro‐inflammatory type). In contrast, no similar phenomenon was observed in Sirt1‐knockout mice [28]. An experimental study on the role of Sesamol in repairing SCI in mice by activating Sirt1 also revealed the profound role of Sirt1 in altering the phenotype of microglia and regulating the inflammatory microenvironment following SCI [51]. Recently, Wu et al. reported that overexpression of Sirt1 in microglia using genetic modification or treatment with Sirt1 agonists could significantly facilitate the phenotype of these cells toward an M2 and suppress the expression of inducible nitric oxide synthase and the pro‐inflammatory mediator interleukin‐1β. This is due to Sirt1 activation leading to reduced expression of acetyl‐p53 and activated STAT1 in microglia [87].

In general, Sirt1 may exert a protective effect against SCI by suppressing neuroinflammation in microglia. This extensive regulatory capacity highlights the potential of Sirt1 as a therapeutic target for SCI treatment by influencing microglial activity.

### 3.5 Sirt1's Impact on Vascular Endothelial Cells

Vascular endothelial cells are the main component of the blood‐spinal cord barrier (BSCB), and the normal maintenance of their function is crucial for the homeostasis of the nervous system [123]. Spinal cord injury often compromises the BSCB, leading to inflammation, neuronal damage, and further tissue deterioration [124, 125]. Knockout of Sirt1 weakens endothelial cell viability in SCI mice, reduces tight junction protein expression (such as ZO‐1, Occludin) by 50%, increases barrier permeability twofold, and significantly damages BSCB integrity and post‐injury tissue repair [29, 126, 127].

Sirt1 is pivotal in upholding the structural and functional integrity of both blood–brain barrier (BBB) and BSCB. In earlier studies using an aging mouse model, it was observed that older mice exhibited a more permeable blood–brain barrier (BBB), and endothelial cells isolated from these mice showed lower levels of Sirt1 expression. Further research using Sirt1 knockout transgenic mice and brain endothelial cell‐specific Sirt1 knockout mice confirmed Sirt1's significant impact on BBB integrity and permeability [128]. The molecular mechanisms underlying BSCB disruption after SCI involve a complex cascade of events. Within hours of injury, mechanical trauma directly damages endothelial cells, disrupting tight junctions and adherens junctions, leading to increased barrier permeability. Studies have shown that the activation of Sirt1 deacetylates and stabilizes key tight junction proteins including ZO‐1, occludin, and claudin‐5, helping maintain their proper localization and function. A recent study by Yin et al. [29] revealed that transgenic mice with endothelial‐specific Sirt1 knockout showed a 50% decrease in tight junction protein expression and a twofold increase in EB extravasation compared with controls after SCI, accompanied by more pronounced intraparenchymal inflammatory cell infiltration and worse behavioral function. Interestingly, the study also discovered that administering SRT1720, a Sirt1 agonist, to mice with SCI significantly enhanced the anti‐oxidative stress capacity of endothelial cells by promoting Sirt1‐mediated deacetylation of p66Shc, attenuating BSCB integrity [29]. Additionally, related in vitro experiments have explored other protective mechanisms of Sirt1 in endothelial cells. A recent study demonstrates that SIRT1 contributes to OGD‐induced endothelial cell survival and angiogenesis, promoting the expression of VEGFA, SIRT1, and BCL‐XL by regulating miR‐377 [89]. Ruan and colleagues reported that Hydroxysafflor Yellow A markedly upregulates the expression of Sirt1 in OGD/R‐injured vascular endothelial cells, and improves endothelial damage by upregulating the downstream HIF‐VEGF pathway [88].

The extracellular matrix (ECM) surrounding BSCB also undergoes dramatic remodeling after SCI, which significantly impacts barrier integrity and neural repair [129]. Within 24 h post‐injury, matrix metalloproteinases (MMP) are dramatically upregulated, leading to degradation of key ECM components like collagen IV and laminin. Studies have revealed that Sirt1 activation reduces MMP expression by 40%–50% through deacetylation of their transcriptional regulators [130, 131, 132]. Additionally, Sirt1 promotes the expression of tissue inhibitors of metalloproteinases (TIMPs), helping maintain ECM homeostasis [133]. This dual regulation contributes to maintain ECM homeostasis. Therefore, the regulation of Sirt1 could potentially preserve BSCB integrity and promote functional recovery after SCI by maintaining a delicate balance between ECM degradation and synthesis.

In conclusion, Sirt1 plays a crucial role in regulating microvascular endothelial cell function, maintaining the integrity of the BSCB, and ameliorating SCI. These regulatory mechanisms provide significant theoretical evidence to support the continued investigation of Sirt1 as a potential therapeutic target for SCI.

## 4 Future Directions for Sirt1 in SCI

**FIGURE 3:**
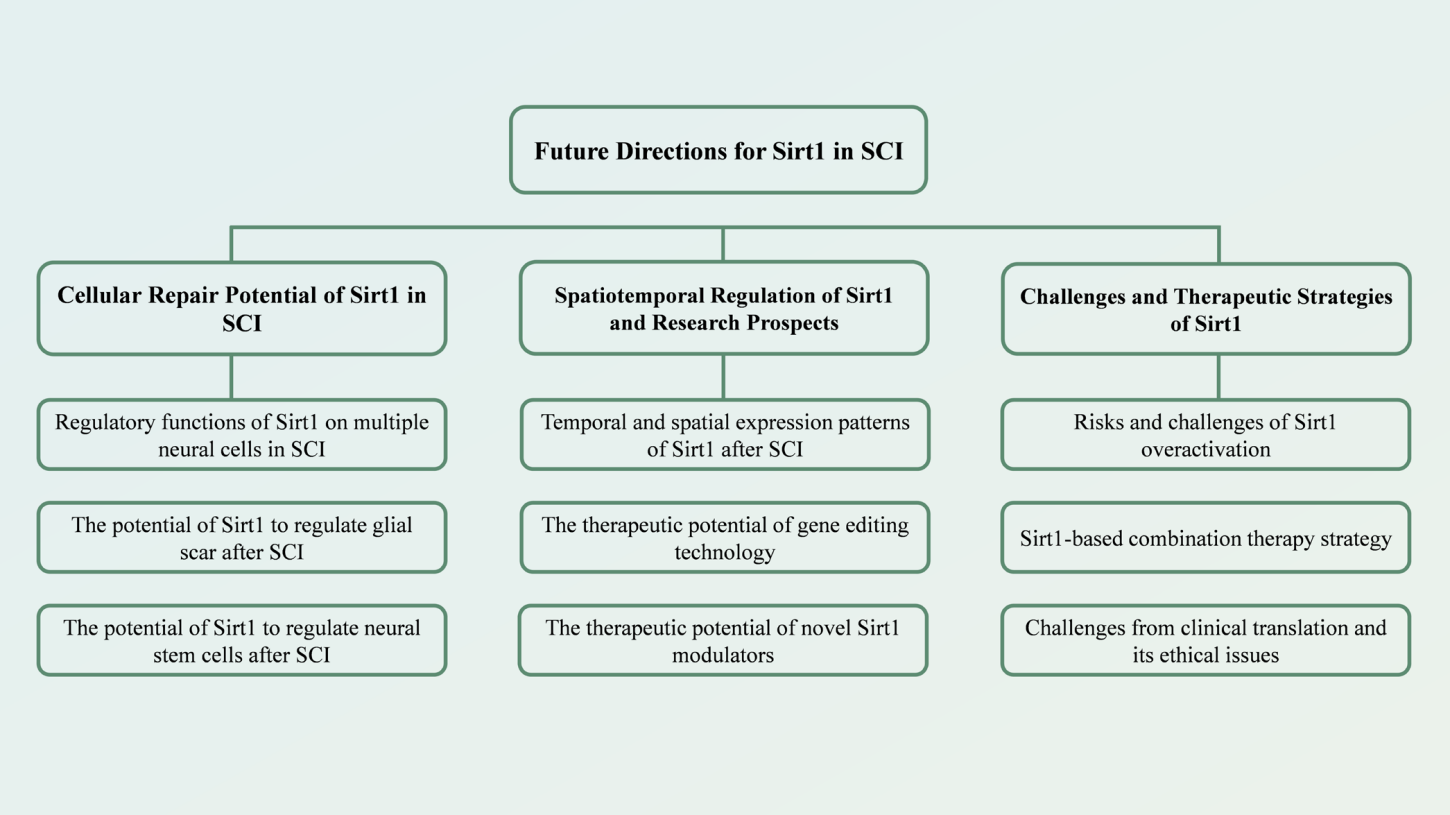
Future directions for Sirt1 in SCI.

In the current study of spinal cord injury, Sirt1 has become a pivotal research object due to its diverse regulatory functions (Table 1). However, the role of Sirt1 in SCI is extremely complex, especially the variations in expression levels observed across distinct cell populations, the spatiotemporal regulation of its activity, and the effects that may be caused by overactivation, all of which bring challenges and opportunities to its application in the treatment of SCI (Figure 3).

**TABLE 1:** Multiple regulatory roles of Sirt1 in SCI.

Intervention strategy	Model system/mechanism	Functions	References
Pharmacological agents			
Melatonin	Mice SCI model/SIRT1‐Drp1	Inhibit apoptosis and protect mitochondria	Zhong et al. [36]
Resveratrol	Rat SCI model/Upregulate Sirt1, LC3‐II, beclin‐1	Enhance autophagy	Tian et al. [62]
Caffeic acid phenethyl ester	Mice SCI model/Activate SIRT1/PGC1α/DRP1	Anti‐neuroinflammation, and improve mitochondrial function	Zhang et al. [34]
Sesamol	Rat SCI model/Activate SIRT1/NF‐ κB	Regulate microglia polarity and reduce neuroinflammation	Feng et al. [51]
Tetramethylpyrazine and astragaloside IV	Rat SCI model/Upregulate Sirt1‐NF‐κB pathway	Regulation of astrocyte A1/A2 polarization	Rao et al. [27]
Oxymatrine	Rat SCI model/Activate SIRT1/AMPK	Enhanced autophagy and inhibit apoptosis	Li et al. [22]
CGPG‐HFn@MnO_2_/AS	Rat SCI model Activate SIRT1, upregulate xCT, GPX4	Alleviate oxidative stress and inhibit ferroptosis	Sun et al. [134]
Minocycline and botulinum toxin	SCI‐Induced NP Rats Model/Activate SIRT1, inactivate NF‐κB, P53, PI3K/AKT	Alleviating SCI‐induced neuropathic pain by inhibiting inflammatory response in astrocytes	Yu et al. [84]
microRNA/extracellular vesicle			
miR‐448	Rat SCI model/Down‐regulate SIRT1	Induce neuronal apoptosis	Wang et al. [135]
miR‐494	Rat SCI model/Suppressed SIRT1/p53	Induce apoptosis	Yu et al. [136]
H‐MSCs‐EVs	Rat SCI model/Activate SIRT1/Nrf2/HO‐1	Anti‐apoptosis and reduced oxidative stress	Rao et al. [137]
EF‐sEVs	Rat SCI model/Upregulate SIRT1/AMPK	Activate autophagy and inhibit apoptosis	Li et al. [35]
Small molecule modulators			
SRT1720	Rat SCI model/Down‐regulate Wnt/β‐catenin	Inhibit microglial activation and anti‐inflammation	Lu et al. [122]
EX‐527	Zebrafish SCI model/Inhibiting the deacetylation of Dnmt1	Inhibiting axon regeneration in Zebrafish	Gupta et al. [25]
HDAC3	Rat SCI model/Upregulate SIRT1/Nrf2 axis	Suppress microglia activation and neuroinflammation	Chen et al. [50]
NFAT5	Astrocyte OGD/R model/Activate of the SIRT1/Nrf2	Prevent astrocyte apoptosis	Xai et al. [119]
DEL‐1	Mice SCI model/Upregulate SIRT1/SERCA2 axis	Inhibiting oxidative stress and alleviating apoptosis	Cheng et al. [23]
Gene therapy			
Endothelial cell‐specific SIRT1 knockout mice	Mice SCI model/High levels of ace‐p66Shc	Promotes oxidative stress in endothelial cells and exacerbates BSCB	Jiang et al. [29]
Overexpression SIRT1	Mice CCI model/Inactivate CaMKIIα^+^ neuron	Alleviate neuropathic pain	Wang et al. [138]
SIRT1 knock‐out	Mice SCI model/Increase acetyl‐NF‐κB p65	Promoting M1 microglial activation aggravates neuroinflammation	Chen et al. [28]
Overexpression SIRT1	Mice EAE model/Upregulation of BDNF and NAD levels	Reduce oligodendrocyte apoptosis and demyelination	Nimmagadda et al. [85]

Abbreviations: CGPG‐HFn@MnO_2_/AS, Chitosan/polyvinyl alcohol/glutaraldehyde/sodium β‐glycerophosphate‐HFn@MnO_2_/Astragaloside IV; DADLE, [D‐Ala^2^, D‐Leu^5^]‐enkephalin; DEL‐1, Developmental endothelial locus‐1; EAE, experimental autoimmune encephalomyelitis; EF‐sEVs, Mesenchymal stem cell‐derived small extracellular vesicles; HDAC3, Histone Deacetylase 3; H‐MSCs‐EVs, Hypoxic‐preconditioned mesenchymal stem cell‐derived small extracellular vesicles; NFAT5, Nuclear factor of activated T cell.

### 4.1 The Cellular Repair Potential of Sirt1 in SCI

Sirt1 exhibits differential effects across various types of neural cells. Recent investigations have substantiated the critical function of Sirt1 in neurons by enhancing their survival and promoting axonal regeneration following SCI [25, 34]. In astrocytes and microglia, Sirt1 has a compelling role in modulating their polarization and mitigating neuroinflammation after SCI [51, 117]. For vascular endothelial cells, the protective mechanism of the blood‐spinal cord barrier after SCI mediated by Sirt1 reveals its critical regulatory role on this cell [29]. Notably, SCI also triggers extensive reactive gliosis, which in turn causes significant scar formation—a critical barrier to axonal regeneration and functional recovery post‐SCI [139, 140]. Research has highlighted that Sirt1 reduces GFAP expression and inhibits astrocyte activation by regulating the MAPK pathway, which potentially mitigating scar formation [141]. Studies have also shown that Caffeic acid phenethyl ester activates the Sirt1‐PGC1α axis, causing a significant reduction in the expression of local GFAP in the tissue of mice with SCI [34]. Consequently, in the future, exploring the relationship between Sirt1, scarring, and neural regeneration may open a new research avenue in the context of SCI. In addition, activating and regulating the differentiation of endogenous neural stem cells (NSCs) into neurons after SCI has been considered a very promising repair strategy. Current studies have shown that *Momordica Charantia* polysaccharides can promote β‐catenin deacetylation by upregulating Sirt1 after cerebral ischemia–reperfusion injury, thereby promoting the transformation of NSC differentiation potential from glial cell lines to neurons [142]. Jian et al. [143] also found that overexpression of Sirt1 could safeguard neural stem cells against camptothecin‐triggered apoptosis via deacetylating histone H3 at lysine 9. Therefore, exploring the mechanism of regulating Sirt1‐mediated NSCs differentiation is expected to become another vital strategy for the endogenous repair of SCI in the future, which may open up new research directions for the search for SCI treatment.

### 4.2 The Spatiotemporal Regulation of Sirt1 in SCI and Future Research Prospects

The regulatory role of Sirt1 in SCI is not only cell‐specific but also complex in terms of spatiotemporal regulation. Sirt1 activity varies significantly at different stages and in different tissue regions after SCI [144]. Current studies have shown that after SCI, Sirt1 protein expression significantly decreased in early stages (8 h–1 day), followed by gradual restoration during subacute and chronic phases, as evidenced by both protein and mRNA levels [29]. This temporal regulation pattern has been observed across multiple neural cell populations—neurons show early Sirt1 reduction followed by a gradual recovery, while astrocytes and microglia show persistent alterations in Sirt1 levels, and these changes are strongly associated with their activation states [27, 28, 51]. Spatially, the decline in Sirt1 expression appears more severe at the injury epicenter compared to the surrounding spinal cord tissue, with especially notable changes also observed in the vascular endothelial cells at the injury site [29]. Although single‐cell sequencing data specifically examining Sirt1 expression patterns after SCI are limited, studies based primarily on immunostaining and protein/RNA analysis have provided valuable insights into Sirt1 spatiotemporal expression patterns, at least to some extent. Therefore, in the acute injury period, the activation of Sirt1 contributes to inhibit oxidative stress and acute inflammatory response, which is crucial for neuroprotection [34]. In the chronic injury period, the activity of Sirt1 is closely related to neural repair and functional recovery [27]. However, premature or delayed activation of Sirt1 may lead to counterproductive outcomes. Specifically, premature Sirt1 activation may disrupt the acute inflammatory cascade through excessive suppression of NF‐κB signaling and pro‐inflammatory cytokines (TNFα, IL‐1β), which are essential for recruiting neutrophils and macrophages to clear cellular debris [145, 146]. This disruption may impair the beneficial aspects of early inflammation, including the release of damage‐associated molecular patterns (DAMPs) that initiate tissue repair programs [147]. Additionally, premature hyperactivation of Sirt1 also leads to excessive deacetylation of FOXO3a, causing an early upregulation of antioxidant enzymes, which overzealously reduces ROS levels. This premature ROS clearance may interfere with the beneficial ROS signaling required for initiating tissue repair, promoting M2 macrophage polarization, and stimulating neural stem cell proliferation [148, 149]. Conversely, delayed activation of Sirt1 could miss the critical therapeutic window for neuroprotection, as the cascade of secondary injury mechanisms, including oxidative stress, calcium overload, and mitochondrial dysfunction, would have already caused substantial neuronal damage. In general, the issue of temporal and spatial regulation necessitates that future designs of Sirt1‐targeted therapies must precisely control the timing and location of its activation to achieve optimal therapeutic outcomes.

Future research should further focus on the spatiotemporal regulation mechanism of Sirt1 in SCI repair, and examine its role across different pathological stages and cell types. Among various promising research directions, two areas warrant particular attention. First, the application of gene editing technologies, particularly CRISPR‐Cas9‐based approaches, holds tremendous potential for manipulating Sirt1 expression in specific cell types after SCI [150]. This technology could enable precise temporal and spatial control of Sirt1 activity, allowing researchers to optimize its therapeutic effects while minimizing potential side effects. Cell type‐specific regulation can be accomplished by using cell type‐specific promoters (such as GFAP promoter for astrocytes, Iba1 promoter for microglia, and Synapsin promoter for neurons) combined with CRISPR‐Cas9 system, while temporal control could be realized through inducible CRISPR systems such as dCas9 fused with light‐sensitive proteins or chemically‐induced proximity (CIP) systems, allowing Sirt1 activation to be controlled by light exposure or small molecule administration at specific time points after injury [151]. Moreover, the development of novel delivery systems, such as AAV serotypes optimized for specific neural cell populations in the spinal cord, could enhance the precision of cell type‐specific Sirt1 regulation [152]. Second, the development of next‐generation Sirt1 modulators represents another crucial avenue for achieving precise spatiotemporal control. Current Sirt1 activators, while promising, often lack specificity and have limited bioavailability in the central nervous system. Several innovative strategies show promise: (1) Temporal control could be achieved through light‐activated Sirt1 modulators (photoswitchable molecules) that can be activated specifically during the optimal therapeutic window after SCI; (2) Spatial specificity could be enhanced by developing tissue‐targeted drug delivery systems, such as spinal cord‐homing peptides conjugated to Sirt1 modulators or injectable hydrogels that provide sustained local release [153]; (3) using cell‐penetrating peptides that specifically recognize surface markers on different neural cell types or developing engineered exosomes expressing cell type‐specific targeting peptides on their surface represents another promising strategy for achieving cell‐selective Sirt1 modulation [154]. Additionally, novel drug delivery platforms such as biomimetic nanoparticles or extracellular vesicles could be engineered to carry Sirt1 modulators specifically to the injury site, thereby minimizing off‐target effects in other tissues [155].

### 4.3 Challenges and Therapeutic Strategies of Sirt1 in SCI Treatment

Although the protective role of Sirt1 in SCI has been widely recognized, its overactivation may bring a series of negative effects that require careful consideration. Key concerns include: (1) Metabolic disruption—excessive Sirt1 activation can lead to uncontrolled deacetylation of metabolic enzymes and transcription factors, disrupting cellular energy homeostasis and potentially triggering metabolic crisis in already stressed neural cells; (2) Inflammatory dysregulation—while Sirt1 activation generally suppresses inflammation, its overactivation during the acute phase may excessively inhibit the beneficial aspects of early inflammatory responses, including the clearance of cellular debris and the release of repair‐promoting factors [156]; (3) Cellular dysfunction—hyperactive Sirt1 can inappropriately deacetylate non‐target proteins, potentially disrupting normal cellular processes and signal transduction pathways. To achieve a balance of Sirt1 activation and maximize its therapeutic benefits while reducing risks, multiple strategies can be integrated into a comprehensive approach. First, in terms of temporal control, pulsed or intermittent Sirt1 activation can be implemented instead of continuous activation, particularly through the use of controllable expression systems such as Tet‐On/Off systems or optogenetic approaches that enable precise temporal regulation [157]. Second, Key downstream effects of Sirt1 activation (such as NAD^+^ levels or key metabolic parameters) can be monitored to adjust activation levels in real time [158]. Finally, in terms of cell‐type specificity, Compounds could be developed that preferentially activate Sirt1 in specific populations of neural cells where the effects are most beneficial.

Besides, exploring combination therapeutic strategies represents another promising direction for optimizing Sirt1‐based treatments in SCI. Several potential approaches warrant investigation: (1) Co‐administration of Sirt1 activators with anti‐inflammatory agents (such as specialized pro‐resolving mediators or IL‐10) could provide synergistic effects in reducing inflammation while promoting tissue repair [122]. (2) Integration of Sirt1 activators with neuroprotective factors (like BDNF or NT‐3) might simultaneously enhance neuronal survival and axonal regeneration [159]. (3) The development of multifunctional nanocarriers that can co‐deliver Sirt1 activators with other therapeutic agents represents an innovative approach. These could include biomimetic nanoparticles designed to cross the BSCB, or injectable hydrogels that provide sustained co‐release of multiple therapeutic agents [134]. The timing and sequence of combination therapies are crucial considerations. Initial administration of anti‐inflammatory agents followed by Sirt1 activators might better align with the natural progression of injury response. Additionally, smart delivery systems responding to the local microenvironment could optimize therapeutic agent release. Future studies should systematically evaluate these combination strategies using standard in vivo SCI model to determine optimal drug combinations, dosing ratios, and administration schedules.

While these innovative approaches show promise, several critical translational barriers must be addressed for successful clinical implementation of Sirt1‐based therapies. The first major challenge lies in effectively delivering Sirt1 modulators across the BSCB. Most current small molecular modulators exhibit limited BSCB penetration due to their molecular characteristics and the barrier's selective permeability [160]. To overcome this challenge, several innovative strategies are worth exploring. We can try to work on the chemical modification of existing Sirt1 modulators through lipidization or the addition of BSCB‐targeting moieties to enhance their BSCB permeability. Meanwhile, developing novel delivery systems, such as lipid nanoparticles or exosomes engineered with BSCB‐penetrating peptides. Additionally, we can implement targeted delivery approaches that utilize receptor‐mediated transcytosis mechanisms naturally present in the BSCB [12, 161, 162]. The second significant challenge involves managing potential off‐target effects of Sirt1 modulation, as Sirt1's widespread expression and involvement in multiple cellular processes throughout the body means that non‐specific activation or inhibition could lead to unintended consequences. To address this issue, we can attempt various approaches including structure‐based drug design for tissue‐specific Sirt1 modulators, targeted delivery systems using spinal cord‐specific biomarkers, and controlled activation strategies with light‐sensitive or chemically‐induced proximity [163]. The third major challenge involves achieving precise spatiotemporal control of Sirt1 modulation in clinical settings. To overcome this challenge, we can attempt temporal control through pulsed delivery systems aligned with specific SCI pathophysiology phases and cell type‐specific delivery using antibody‐conjugated nanoparticles targeting neural cell surface markers. Additionally, implementing real‐time monitoring systems to measure local Sirt1 activity biomarkers (e.g., NAD^+^/NADH ratio, acetylation levels of target proteins) could help optimize the timing and dosing of interventions [158]. These approaches need to be validated in preclinical models before translation, with careful consideration of the technical feasibility and regulatory requirements for clinical implementation.

Moreover, ethical considerations and safety concerns regarding long‐term Sirt1 activation warrant careful attention. This may promote uncontrolled cell survival and proliferation through sustained activation of pro‐survival pathways and suppression of tumor suppressors, particularly through its interactions with p53 and FOXO family proteins, potentially increasing cancer risk [164, 165]. The sustained deacetylation activity of Sirt1 could also lead to extensive epigenetic modifications, resulting in lasting changes in gene expression patterns with unpredictable long‐term consequences on cellular function and tissue homeostasis. Additionally, given Sirt1's widespread expression throughout the body and its involvement in multiple physiological processes including metabolism, stress response, and aging, the off‐target effects of Sirt1 in non‐neural tissues deserve attention [166, 167]. Systemic Sirt1 activation may potentially affect multiple organ systems, which could be particularly concerning in the context of long‐term therapeutic interventions.

Sirt1 shows significant therapeutic potential in SCI treatment but faces multiple challenges. Future research directions primarily encompass three aspects: (1) Further exploration of Sirt1's repair potential in different neural cells, particularly its role in neural stem cell differentiation and scar formation; (2) Optimization of spatiotemporal regulation strategies for Sirt1, including the development of novel delivery systems and precise activation mechanisms; (3) Addressing the adverse effects of Sirt1 overactivation and investigating combination therapeutic approaches. These studies will provide a crucial theoretical foundation and practical guidance for developing more effective SCI treatment strategies.

## 5 Conclusion

This article reviews the multiple regulatory roles of Sirt1 in SCI repair in detail. Existing studies have shown that Sirt1 plays a core role in neuroprotection and tissue repair by regulating inflammatory response, oxidative stress, autophagy, and mitochondrial function. Sirt1 can mediate the functional regulation of neurons, astrocytes, microglia, oligodendrocytes, and vascular endothelial cells through multiple signaling pathways, promoting axon regeneration, myelin repair, and maintenance of the BSCB after SCI. However, the spatiotemporal expression of Sirt1 and its differential effects in different cell types increase the complexity and challenge of its application in SCI treatment. Therefore, future research should focus on the intricate regulatory mechanisms of Sirt1 in SCI, particularly its role across different pathological stages and specific cell types, with the aim of providing a theoretical foundation and practical guidance for the development of novel therapeutic strategies for SCI.

## Author Contributions

J.L. and F.J. designed the manuscript. J.L., S.C., and Y.L. drafted the manuscript. C.Z. and F.J. carefully reviewed the manuscript. All authors approved the final version of the manuscript.

## Conflicts of Interest

The authors declare no conflicts of interest.

## Data Availability

The data that support the findings of this study are available from the corresponding author upon reasonable request.
